# Competing strain and resource pathways linking AI-enabled work systems to employee wellbeing

**DOI:** 10.3389/frai.2026.1922774

**Published:** 2026-09-09

**Authors:** A. S. Sathish, R. Indradevi

**Affiliations:** 1 VIT Business School Vellore Institute of Technology (VIT), Vellore, India; 2 Department of Commerce, School of Social Sciences and Languages, Vellore Institute of Technology (VIT), Vellore, India

**Keywords:** artificial intelligence, digital work intensity, employee wellbeing, perceived organisational support, psychological resilience, technostress, workplace digitalisation

## Abstract

**Purpose:**

With the growing integration of Artificial Intelligence (AI) into contemporary workplaces, understanding how AI-enabled work systems influence employee wellbeing has become an important concern within HRM research, digital work literature, and future-of-work scholarship.

**Design/methodology/approach:**

Drawing on the Job Demands–Resources (JD-R) model and Conservation of Resources (COR) theory, this study examines the mediating roles of technostress and psychological resilience in the relationships between AI adoption, digital work intensity, and employee wellbeing, while also testing the moderating role of perceived organisational support. Data were collected from 516 employees in the Indian IT industry and analysed using Partial Least Squares Structural Equation Modelling (PLS-SEM).

**Findings:**

The findings suggest that the direct effect of AI adoption on employee wellbeing remains unclear, while digital work intensity positively influences wellbeing. Both AI adoption and digital work intensity significantly affect technostress and psychological resilience, reflecting the dual nature of AI-enabled work environments. Technostress does not significantly influence employee wellbeing, whereas psychological resilience is a significant predictor and mediator. Perceived organisational support was positively associated with employee wellbeing; however, its moderating effect on the relationship between technostress and employee wellbeing was not significant.

**Originality/value:**

The study is original because it shows that AI-based work systems create both strain and resource-building processes. The results also indicate that psychological resilience has a stronger influence on employees’ psychological responses than technological strains. The paper offers a fresh perspective on employees’ psychological responses, digital transformation, and the impact of AI on the human factor in technology-rich work environments.

## 1 Introduction

Artificial Intelligence (AI) is rapidly becoming part of the modern work environment, revolutionising the way people work, think, and use technology. The integration of AI into work systems can improve productivity, efficiency, and innovation, while also creating new job demands, such as increased cognitive load, continuous learning requirements, and greater job complexity. This has brought the implications of AI for employee wellbeing into focus as a key theme of HRM, digital work and future-of-work research.

In the second generation of workplace digitalisation, AI systems are not just automating laborious tasks and making information transfer more efficient. Still, they are now involved in cognitive tasks like decision support, content creation, predictions, recommendations, and algorithmic management. The people who work with the equipment no longer only work with digital devices, but now work with intelligent systems that affect how tasks are done, decisions are made, and the work process is carried out. This shift changes the nature of workers from technology users to human–AI collaborators, introduces new psychological demands like algorithmic uncertainty, ongoing skill development, diminished autonomy in tasks, and increased reliance on AI-generated outputs, and offers opportunities for workers to learn, create, and make decisions more effectively. AI-based work systems are therefore a qualitatively different type of work system than are traditional digital technologies.

Previous studies have shown positive and negative impacts of AI and digitalisation in the workplace. On the other hand, AI technologies can enhance work efficiency and decision quality and provide learning opportunities. However, they can also lead to technostress, cognitive overload and work-related strain. While these positive and negative effects are well documented, much less research has been conducted on the psychological processes by which such work systems affect workers’ wellbeing.

Existing studies have either focused on the stress resulting from the use of AI in the workplace (also known as technostress) or on the productivity, learning, and performance gains of AI use alone. Thus, little is known about employees’ experiences of using AI in both capacities as a technological demand and as a valuable aspect of work, while simultaneously doing their job in the same working environment. This has led to a lack of clarity regarding the conflicting psychological processes that operate between AI-powered work systems and employee wellbeing. Moreover, previous research has focused on either the phenomenon of technostress or the concept of psychological resilience, offering only limited insights into the interplay between process-based and resource-based approaches in technostress. Also, previous studies predominantly examined technostress or psychological resilience alone and thus offered only partial answers to the question of how technostress and psychological resilience process together. Further, the use of perceived organizational support as an organizational resource that influences employees’ reactions to AI-based work requirements is under-researched.

To fill these gaps, this study utilises the Job Demands–Resources (JD-R) model and Conservation of Resources (COR) theory to investigate technostress and psychological resilience as opposing psychological pathways between the adoption of AI and digital work intensity, as well as between digital work intensity and employee wellbeing, and to test the moderating effect of perceived organisational support (POS). The study also contributes to understanding how AI-powered work systems affect workers’ wellbeing by embedding these mechanisms within a comprehensive framework, rather than merely cataloguing the positive and negative effects of workplace AI.

## 2 Theoretical framework

### 2.1 Job Demands–Resources (JD-R) model

“The Job Demands–Resources (JD-R) perspective offers a theoretical basis for examining employee responses to work characteristics in new work contexts. It posits job demands, which demand sustained cognitive or emotional effort and lead to psychological costs, and job resources, which help achieve goals, develop personal capacities and enhance wellbeing.

The AI-enabled work systems are conceived as generating multiple job demands and multiple job resources at the same time as AI is being used, based on the JD-R model. As with all digital tech, AI presents some unique job demands that vary from those of traditional digital tools, such as the need for new skills in and around AI, uncertainty over AI-driven recommendations, evolving job requirements, enhanced cognitive demands, and the ability to work alongside AI intelligence. These demands involve constant mental exertion and can cause stress. Simultaneously, AI can serve as a valuable tool for the workforce, streamlining repetitive tasks, aiding decision-making, learning, and problem-solving, and allowing staff to concentrate on more significant tasks. This results in a dual workplace environment where workers face both technology-related demands and opportunities.

In this context, technostress reflects the process of health impairment, in which excessive digital demands lead to cognitive and emotional resource depletion, potentially resulting in health impairment. By contrast, psychological resilience represents a motivational process that allows people to draw on their own resources, re-appraise stressors, and maintain optimal functioning in the face of digital demands.

Accordingly, the JD-R model suggests that employees’ responses to AI-enabled work systems emerge from the simultaneous interaction of these competing demands and resources. Rather than viewing AI solely as a source of stress or productivity enhancement, the framework explains how employees continuously balance AI-related pressures with opportunities for learning, adaptation, and performance improvement.

### 2.2 Conservation of Resources (COR) theory

The Conservation of Resources (COR) theory adds dynamism to the JD-R model by explaining how people respond to gains and losses of resources in stressful situations. According to COR theory, people seek to gain, maintain, and protect resources they value, with stress resulting from the loss or threat of these resources.

In the context of AI-enabled work systems, technostress is not limited to digital overload, but also refers to work demands imposed by AI. Workers need to constantly learn new skills in the realm of AI, understand and confirm AI-generated suggestions, adjust to new and changing roles, and understand how to work effectively with intelligent systems, while also learning to handle uncertainty around algorithmic outputs. Such tasks are cognitively and emotionally demanding and drain people’s psychological resources, which can lead to poorer wellbeing.

In turn, exposure to challenging digital environments can lead to gains in resources, particularly psychological resources. According to COR theory, exposure to workplace challenges may contribute to the development of psychological resources. From the perspective of COR theory, these AI-enabled work demands consume valuable cognitive and psychological resources. When employees perceive that the effort required to adapt to AI exceeds the resources available to them, technostress emerges, increasing the likelihood of resource loss and psychological strain.

Within AI-integrated work systems, psychological resilience is defined as an ongoing personal resource shaped by employees’ engagement with intelligent technologies. The more employees become familiar with AI recommendations, deal with uncertainty, adjust their roles to embrace new responsibilities, and work well with AI, the more confident, adaptable, and able to cope they feel. A COR perspective on these adaptive experiences is that they are resource-building experiences—they help employees preserve and restore their psychological resources, and they help employees maintain their wellbeing despite the challenges of AI-enabled work.

While resource gain spirals are theoretically plausible, the present study does not directly examine changes in resources over time.

COR theory proposes that resource gains and losses may occur simultaneously. However, the present study examines these processes from a cross-sectional perspective and therefore does not test dynamic resource gain or loss over time.

### 2.3 Combining JD-R and COR in digital environments

The Job Demands–Resources (JD-R) model and Conservation of Resources (COR) theory provide complementary perspectives for understanding employees’ psychological responses within AI-enabled work systems. The JD-R model emphasises the role of workplace demands and resources in shaping employee outcomes. At the same time, COR theory centres on how problems and changes influence employees’ attempts to gain, sustain, and defend valued resources (Hobfoll, 2001; Bakker and Demerouti, 2007).

When applied to an AI-powered workplace, these perspectives imply that workers face technology-based challenges and opportunities to develop resources simultaneously. The use of AI and digital work intensity can introduce complexity, uncertainty, and lifelong learning challenges, all of which are components of technostress. The simultaneous exposure to technology-rich work contexts can also promote the development of adaptive resources, such as psychological resilience. Strain-based and resource-based mechanisms will likely interact to influence employee wellbeing.

Based on this integrated perspective, the present study conceptualises technostress as the strain pathway through which AI adoption and digital work intensity deplete employees’ psychological resources. In contrast, psychological resilience represents the resource pathway that enables employees to adapt successfully to AI-enabled work environments and maintain their psychological wellbeing. Furthermore, the study incorporates perceived organisational support as an organisational resource that moderates employees’ responses to AI-enabled work demands by buffering the adverse effects of technostress and facilitating adaptation to AI-driven workplaces.

The study integrates the JD-R model and COR theory and conceptualises AI adoption and the intensity of digital work as traits of AI-enabled work systems that simultaneously create job demands and opportunities for resource development. Digital work intensity is mainly a job demand that raises workload, cognitive effort and continuous connectivity. Like most other technologies, AI comes with its own learning curves, technical challenges, and uncertainties, as well as opportunities for increased efficiency and skill development. Technostress is then defined as a strain reaction to these job demands, while psychological resilience is a personal resource that facilitates an employee’s appropriate response to technological demands. Perceived organisational support is considered an organisational job resource, offering employees support, guidance, and access to digital transformation resources. The focus outcome is employee wellbeing, which represents psychological functioning in AI-supported work. Thus, this study is novel in showing that participants interacting with AI work systems simultaneously experience resource-draining technostress and resource-building psychological resilience. These competing pathways do not exist in isolation, but instead work in tandem to explain employee wellbeing in AI-enabled workplaces in a more holistic manner than investigations that study employees’ strain or benefit from AI, or studies that investigate the benefits of AI.

Accordingly, the JD-R model explains how AI-enabled work systems simultaneously generate job demands and job resources. In contrast, COR theory explains how employees lose psychological resources through technostress while simultaneously building personal resources through psychological resilience as they adapt to intelligent work systems. Their integration therefore provides a comprehensive explanation of employee wellbeing in AI-enabled workplaces.

## 3 Literature review and hypothesis development

### 3.1 AI in the workplace and digital transformation

Recent research on workplace digitalisation has emphasised that AI implementation should be examined not only as a technological initiative but also as a process requiring organisational and employees’ psychological responses. As digital transformation efforts continue to grow, a crucial aspect is employees’ willingness and ability to interact with smart systems, integrate AI into their workflows, and learn the new skills required for AI-supported work (Venkatesh, 2022; Tursunbayeva and Chalutz-Ben Gal, 2024). Findings from generative AI adoption research show that, when integrating generative AI into current work routines, it is essential to consider not only technological capabilities but also compatibility with existing routines, employee acceptance, and psychological processes (Dwivedi et al., 2023; Nawaz et al., 2024). AI is thus not just a technology but a paradigm shift that will transform the nature of work, job roles, and work practices (Jarrahi et al., 2023).

Additionally, recent research has emphasised the increasing significance of human–AI interaction in today’s work environments. Many AI systems are not intended to replace their human counterparts, but rather to work alongside them to complete tasks, make decisions, or solve problems (Dellermann et al., 2019). Studies show that AI can augment, not replace, human employees, improving their productivity, decision-making capabilities, and task performance (Raisch and Krakowski, 2021). Human–AI collaboration has emerged as a key focus in future-of-work and workplace digitalisation research, highlighting the need to understand how employees engage with, trust, and adapt to AI tools in their daily work activities (Jarrahi et al., 2023).

Algorithmic management represents a fundamental shift in the governance of work, as intelligent systems increasingly mediate tasks handled by human managers (Kellogg et al., 2020). All these technologies could raise new concerns about monitoring, autonomy, transparency, fairness, and employee control (Meijerink et al., 2021). Thus, employees must continually adjust to changing forms of technology-managed work.

### 3.2 Adoption of AI and digital work intensity

The integration of artificial intelligence (AI) and work intensity in the digital age is a critical characteristic of today’s work, especially in the IT industry. The adoption of AI technologies such as machine learning and automation improves efficiency, decision-making, and innovation, enabling companies to streamline operations and build capabilities (Gabriel, 2020; Pan et al., 2022; Kludacz-Alessandri et al., 2025). Meanwhile, the growing use of digital technologies has intensified work through continuous connectivity, multitasking, and faster information processing (Menon et al., 2020; Yan et al., 2025).

In the present study, AI adoption is defined as employees’ integration of AI-enabled tools into their daily work through intelligent automation, AI-assisted decision support, and human–AI collaboration. It reflects the extent to which employees routinely interact with AI systems as part of their work processes, rather than merely the organisational implementation of AI technologies or employees’ perceptions of AI (Lada et al., 2023). In contrast, digital work intensity refers to the pace and volume of digitally mediated work (Marsh et al., 2024), characterised by continuous connectivity, increased information processing, multitasking, and sustained digital engagement. These constructs represent distinct dimensions of AI-enabled work systems and are therefore modelled as separate predictors in the proposed framework.

However, the introduction of AI and increased digital Intensity of work comes with adaptive challenges. Workers are expected to continually learn new technologies, navigate constant change, and process a large volume of digital interactions. This can lead to cognitive overload, time constraints, and work-life conflicts, which affect employee wellbeing (Marsh et al., 2024). Existing research shows this ambivalence in digital work (Marsh et al., 2024; Menon et al., 2020), where technology environments can boost performance while inducing psychological strain (Menon et al., 2020).

### 3.3 Technostress as a strain mechanism

Technostress refers to the strain experienced when employees struggle to adapt to technology-related demands, including overload, complexity, uncertainty, and constant connectivity (Tarafdar et al., 2019; Ragu-Nathan et al., 2008; La Torre et al., 2020).

Research shows that technostress is associated with negative psychological consequences, such as emotional exhaustion, job dissatisfaction and reduced wellbeing (Kim et al., 2016; Rodríguez-Modroño, 2022; Nastjuk et al., 2024). In addition, high levels of digital work intensity exacerbate technostress through cognitive load and frequent interruptions (Rebelo et al., 2024; Rademaker et al., 2025; La Torre et al., 2020). Yet recent studies show that technostress may have different effects across contexts, underscoring the need for a more nuanced understanding of its impact on wellbeing.

### 3.4 Psychological resilience as an adaptive resource

In AI-enabled work systems, psychological resilience is an adaptive resource that helps employees successfully adapt to intelligent technologies (Liao et al., 2022).

Psychological resilience represents an adaptive resource that enables employees to cope effectively with technological change and workplace challenges (Liao et al., 2022). Within AI-enabled work systems, resilience supports learning, flexibility, and positive employee wellbeing in response to intelligent technologies (Nwankpa and Datta, 2017; Gans, 2023).

Research shows that resilience helps buffer against negative impacts of stress and contributes to positive outcomes, such as engagement, satisfaction and wellbeing (Guest, 2017; Nimrod, 2018; Radhakrishnan and Chattopadhyay, 2020). Crucially, resilience is dynamic; challenging experiences and positive work environments foster it (Xintian and Peng, 2023). Consequently, despite the challenges, digital work contexts may be environments for building adaptive capacity, allowing employees to transform digital work challenges and opportunities (Craig and Kuykendall, 2019; Chen et al., 2024).

### 3.5 Wellbeing in the digital environment

Employee wellbeing is seen as employees’ positive psychological functioning and the quality of their work experiences in the technology-mediated environment. The interplay between strain and resource processes determines wellbeing in the digital workplace. Technostress can affect employees’ functioning, whereas psychological resilience can facilitate positive adaptation and employee wellbeing (Upadhyaya and Vrinda, 2021; Nimrod, 2026). It is therefore important to understand the interaction between these mechanisms to explain employees’ outcomes in AI-enabled work systems.

### 3.6 Perceived organisational support

Perceived organisational support is not only about support from superiors and colleagues, but also about AI-specific practices in the organisation, such as structured training programmes for AI, AI technical support, communication about AI implementation, participation in AI decision-making, ethical and fair AI governance, and opportunities for developing AI-related competence (Hamid and Kundi, 2025). This helps improve employees’ confidence in using AI systems and offers organizational tools to help them thrive in an AI-driven workplace.

### 3.7 Research gap

While existing studies have examined the influence of digital technologies on employee outcomes, three areas remain under-researched. First, while prior research has focused on the negative (e.g., technostress) or positive (e.g., performance) effects of digital technologies, few studies have examined these effects within a single framework (Kooij et al., 2013; Tursunbayeva and Chalutz-Ben Gal, 2024). Second, the joint mediating effects of technostress and resilience have not been examined, thereby restricting our understanding of the psychological mechanisms of digital work. Third, there is a need for more research on the moderating role of perceived organisational support in digital work settings.

### 3.8 Hypotheses development

The research draws on the Job Demands–Resources (JD-R) and Conservation of Resources (COR) theories to explore the effects of adopting AI and increased digital work intensity on employee wellbeing through the pathways of demands and resources.

While there is a correlation between positive and negative impacts of AI, such as improved productivity and decision-making quality, and employee wellbeing, there is not yet conclusive evidence of AI’s direct effect on employee wellbeing (Gabriel, 2020; Pan et al., 2022; Neumann et al., 2024). However, some research indicates that the impact of AI is largely indirect, affecting employees through shifts in their job descriptions, working conditions, and adjustment strategies (Page and Vella-Brodrick, 2013). Existing research provides mixed evidence regarding the direct influence of AI adoption on employee wellbeing, suggesting that the relationship remains theoretically ambiguous and warrants empirical examination.

Psychological resilience is also expected to function as an adaptive mechanism through which employees respond to technology-related work demands. Employees exposed to AI-enabled work systems and intensified digital work may develop or draw upon personal resources that help them cope with changing work requirements and maintain positive psychological functioning (). Previous research has demonstrated that psychological resilience can mediate the relationship between occupational stress and quality-of-life outcomes, suggesting that resilience serves as an important psychological pathway through which stressful work conditions influence well-being (Yan et al., 2022; Sahadev et al., 2024).

*H1*: AI adoption is positively associated with employee wellbeing.

Digital work intensity, with ubiquitous connectivity, multitasking, and high information-processing speed, facilitates flexibility and efficiency but also leads to overload and work-life boundary issues (Yan et al., 2025). From the JD-R model standpoint, these can be challenging demands that contribute to engagement and wellbeing.

*H2*: High digital work intensity is positively associated with employee wellbeing.

AI implementation leads to techno-overload, techno-complexity and techno-uncertainty, demanding adaptation (Ragu-Nathan et al., 2008; Salo et al., 2022; Neumann et al., 2024). Likewise, digital work intensity increases interruptions, information overload, and accessibility (Xanthopoulou et al., 2012; Rebelo et al., 2024; Rademaker et al., 2025).

*H3*: AI adoption is positively associated with technostress.

*H4*: Digital work intensity is positively associated with technostress.

However, exposure to an AI-enabled environment and digitally intensive work also promotes learning, adaptability, and skill development, thereby fostering psychological resilience (Nwankpa and Datta, 2017; Craig and Kuykendall, 2019; Gans, 2023; Chen et al., 2024). Challenging work conditions can increase psychological resilience when successfully addressed.

*H5*: AI adoption is positively associated with psychological resilience.

*H6*: Digital work intensity is positively associated with psychological resilience.

Technostress leads to anxiety, fatigue, and reduced wellbeing, whereas psychological resilience supports coping, stability, and wellbeing (Guest, 2017; Nimrod, 2018; Rodríguez-Modroño, 2022). These are opposite processes through which digital work experiences affect employees.

*H7*: Technostress is negatively associated with employee wellbeing.

*H8*: Psychological resilience is positively associated with employee wellbeing.

Drawing on JD-R and COR theory, technostress and psychological resilience are expected to operate as key mechanisms through which AI adoption and digital work intensity influence employee wellbeing. Accordingly, the following mediation hypotheses are proposed.

Technostress may also function as an intervening mechanism rather than merely representing a direct source of strain. From an appraisal and coping perspective, technology-related demands can trigger different cognitive and coping responses, which subsequently influence employee outcomes (Zhao et al., 2020). This suggests that the effects of AI adoption and digital work intensity on employee well-being may operate in part through employees’ experiences of technostress.

*H9*: Technostress mediates the relationship between AI adoption and employee wellbeing.

*H10*: Technostress mediates the relationship between digital work intensity and employee wellbeing.

*H11*: Psychological resilience mediates the relationship between AI adoption and employee wellbeing.

*H12*: Psychological resilience mediates the relationship between digital work intensity and employee wellbeing.

Perceived organisational support encompasses contextual factors such as training, flexibility, and recognition that enhance employee wellbeing (Xu et al., 2022; Aboramadan et al., 2022; Hamid and Kundi, 2025; Kim et al., 2016). It is frequently defined as a moderating variable that buffers against the harmful impact of stress (Bukhari and Kamal, 2015; Al Karim et al., 2025), but it may not have the same effect in high-tech workplaces, where personal resources are more important (Guan et al., 2014; Aboramadan et al., 2022).

*H13*: Perceived organisational support moderates the relationship between technostress and employee wellbeing, such that the negative effect is weaker at higher levels of support.

### 3.9 Conceptual framework

**Figure 1:**
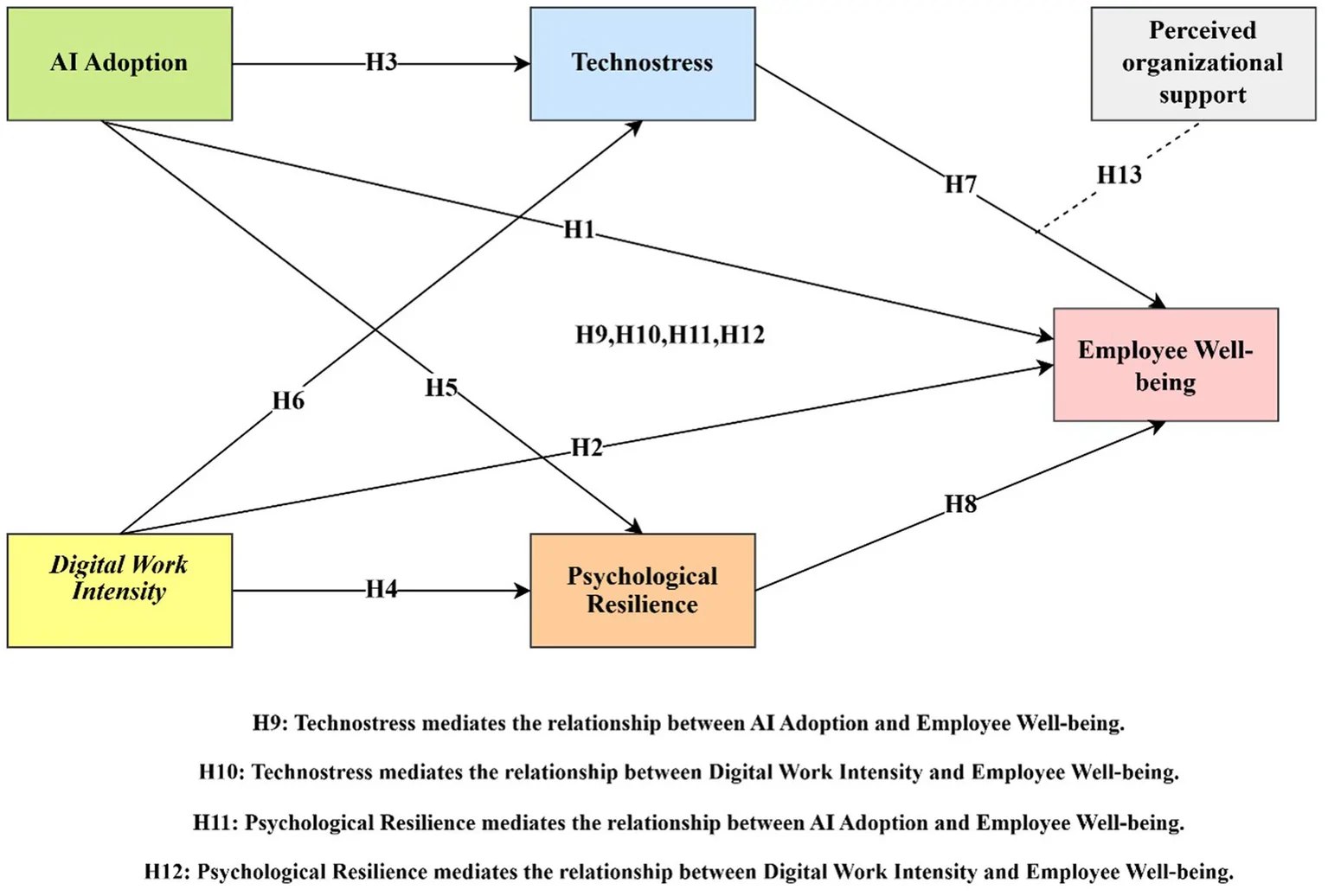
Conceptual framework.

“The conceptual model hypothesises that AI Adoption and Digital Work Intensity are antecedents of Employee Wellbeing, with technostress and psychological resilience mediating the relationship. Digital work intensity and AI adoption should increase technostress while also improving resilience. Technostress is postulated to reduce wellbeing, and psychological resilience is thought to mitigate it. Moreover, Perceived Organisational Support (POS) is introduced as a moderating variable that influences the strength of the relationship between technostress and employee wellbeing. This model offers a balanced view that combines the strain-inducing and resource-enhancing effects of online working conditions on employee wellbeing. Figure 1 shows the conceptual model of the study, illustrating the proposed relationships among AI adoption, digital work intensity, technostress, psychological resilience, perceived organisational support, and employee wellbeing.

## 4 Methods

### 4.1 Context of the study

The Indian IT sector is an apt empirical context to explore the wellbeing of employees working in AI-enabled work systems, as it is one of the largest and fastest-growing digital knowledge industries in the world. India has become a leader in software development, digital services, business process management, and AI-powered technology solutions. This context is also strategically significant, as evidenced by recent industry data. As per the NASSCOM AI Adoption Index (2025), about 87% of Indian enterprises have used AI in at least one business function (Stanford University, 2025). India is also ranked 3rd in the world in the Stanford AI Vibrancy Index. It has around 2.5 times the global average in AI skills penetration, reflecting the country’s strong capabilities in the AI workforce. Moreover, Deloitte India’s *State of AI in the Enterprise* reports that 40% of Indian organisations have achieved significant or full-scale AI implementation, compared with a global average of approximately 28%, indicating that Indian enterprises are moving beyond AI experimentation towards enterprise-wide deployment (Deloitte India, 2025). The developments indicate that the adoption of AI in India is not just experimental, but has moved to become an integral part of work systems across the Indian IT sector, serving as a potentially suitable context to study the psychological reactions of employees to AI-based work systems.

AI-powered work systems, particularly with the rapid adoption of generative AI technologies, are increasingly involved in cognitive tasks such as code generation, decision support, content creation, predictive analytics, workflow optimisation, and algorithm-based decision making, in contrast to traditional digital systems that primarily automate repetitive tasks. As a result, employees in Indian IT organisations are not just users of technology, but partners with intelligent systems that impact how tasks are performed, designed, and decisions are made. This transformation demands new skills, a critical perspective on AI-generated content, knowledge of new AI applications, and the ability to adapt to a constantly changing technology landscape. Recent workforce reports also suggest that AI has been changing job roles and skill sets in the Indian IT industry. The requirement for AI professionals will surge from around 600,000–650,000 to over 1.25 million by 2027, according to a joint Deloitte–NASSCOM (2025) report, and this growth will necessitate ongoing AI upskilling and reskilling (Deloitte & NASSCOM, 2025). Thus, workers are expected to engage with intelligent systems and constantly enhance their analytical, technological, and collaborative capabilities to excel in human–AI collaboration.

These features lead to a work context that also contributes significantly to job demands and provides valuable work resources. Workers often have to engage in ongoing reskilling, deal with more complex thinking, less clarity about algorithms, information overload, and greater expectations for productivity. These changes are also part of a broader trend in job design. Workers are increasingly expected to validate AI output, tweak AI prompts, check AI algorithm recommendations, etc., and use human judgment in making decisions. AI-driven work therefore provides a perfect context for exploring both strain and resource pathways outlined in the JD-R model as well as the Conservation of Resources theory. Meanwhile, AI technologies offer learning and creativity opportunities, decision support, routine activity automation and professional development. The Indian IT industry provides an ideal context for analyzing the competing pathways and resources of the Job Demands–Resources (JD-R) model and the Conservation of Resources (COR) theory since workers are exposed to both strains and resources at the same time.

Moreover, overall, Indian IT workers are relatively digitally competent and regularly interact with work systems that utilise AI. This context allows for assessing how employees view AI-related demands as a stressor or challenge and how it affects psychological resilience and wellbeing. Thus, the IT sector in India offers a theoretically suitable context for examining the potential interplay among psychological pathways and employee wellbeing from AI-driven work systems.

Accordingly, employees in the Indian IT industry routinely engage with AI-enabled work systems, making them an appropriate population for examining how AI-generated job demands and resources jointly influence employee wellbeing.

### 4.2 Data collection and sampling technique

A quantitative research approach was chosen to explore the relationships among AI adoption, digital work intensity, technostress, psychological resilience, perceived organisational support, and wellbeing. The research was conducted over 3 months (December 2025–March 2026) through an online survey.

Participants were selected from 18 medium and large IT and IT-enabled service firms located in Bengaluru, Chennai, Hyderabad, Pune, and the National Capital Region of India. Initial organisational access was obtained through professional networks, alum contacts, HR managers, and LinkedIn-based outreach. Data were collected using an online survey administered through Google Form, and survey invitations were distributed via HR email circulation lists, professional LinkedIn groups, and employee referral networks (snowball sampling).

A total of 1,120 invitations were distributed across the participating firms. Of these, 642 employees completed Wave 1, yielding an initial response rate of 57.3%. Five hundred seventy-one employees completed Wave 2, and 548 responses were successfully matched across the two waves using self-generated identification codes. Following data screening, 32 matched responses were excluded due to substantial missing data (more than 10% unanswered items), straight-lining response patterns, duplicate submissions, inconsistent identification codes, or completion times below one-third of the median survey duration. The final sample consisted of 516 valid matched responses, corresponding to an effective usable response rate of 46.1% of all invited participants.

To be eligible for participation, respondents were required to (1) be employed full-time in an IT or IT-enabled services organisation, (2) have at least 6 months of organisational tenure, (3) use digital work platforms daily, and (4) have direct experience using AI-enabled tools or systems in their work (e.g., generative AI assistants, automated analytics platforms, machine-learning-based decision support systems, or workflow automation tools) for a minimum of 3 months before the survey. Respondents who indicated no direct AI-use experience were screened out at the beginning of the questionnaire and were not allowed to proceed.

The data-cleaning procedure involved multiple stages. First, responses with excessive missing values were removed. Second, duplicate IP addresses and duplicate identification codes were checked and eliminated. Third, response quality was assessed using attention-check items and long-string analysis to identify straight-lining behaviour. Fourth, multivariate outliers were examined using Mahalanobis distance (*p* < 0.001), resulting in the removal of 11 cases. Finally, completion times were inspected, and responses completed in less than 6 min were excluded as insufficient-effort responses.

A formal *a priori* power analysis was conducted using G*Power 3.1 for multiple regression with a medium effect size (*f*^2^ = 0.15), statistical power of 0.95, alpha level of 0.05, and a maximum of six predictors for the most complex endogenous construct. The analysis indicated a minimum required sample size of 146 participants. In addition, the 10-times rule for PLS-SEM suggested a minimum of 60 observations. The final sample of 516 participants therefore provided substantial statistical power for detecting direct, mediating, and moderating effects.

Because employees were nested within organisations, we also assessed the possibility of clustering effects. The average cluster size was 28.7 employees per firm. We calculated intraclass correlation coefficients (ICC1) for the main endogenous construct (employee wellbeing), which ranged from 0.018 to 0.041, indicating that only a small proportion of variance was attributable to between-organisation differences. The corresponding design effect was 1.07, which is below the commonly used threshold of 2.0 for problematic clustering. Accordingly, the data did not exhibit substantial organisational dependency, and single-level PLS-SEM was considered appropriate. We have added these details to the revised Sampling and Participants subsection and reported the power analysis and clustering diagnostics in the methodology section for full transparency.

The sample included employees of IT and IT-enabled services firms in India with significant exposure to digital work and AI. Purposive sampling was employed to ensure participation by those with experience using AI systems, and snowball sampling was used to reach a larger population of professionals via online networks. The study employed a time-lagged two-wave survey design rather than a purely cross-sectional design. Data were collected in two phases from employees working in Indian IT and IT-enabled service firms. Wave 1 was conducted between 20 December 2025 and 10 January 2026, during which data on AI Adoption, Digital Work Intensity, and demographic variables were collected. Wave 2 was conducted between 25 January 2026 and 15 February 2026, approximately 4 weeks after Wave 1, and included measures of Technostress, Psychological Resilience, Perceived Organisational Support, and Employee Wellbeing. To match responses across the two waves while maintaining participant anonymity, respondents generated a self-created identification code based on a predefined combination of letters and numbers. A total of 642 responses were obtained in Wave 1 and 571 responses in Wave 2. After matching the identification codes, 548 responses were successfully paired across both waves. Subsequently, 32 cases were removed due to incomplete or inconsistent responses, resulting in a final analytical sample of 516 matched responses used for the PLS-SEM analysis. The attrition rate from Wave 1 to the matched sample was 14.6%, which is considered acceptable for organisational survey research. We also conducted an early-versus-late respondent comparison, and no significant differences were observed (*p* > 0.05), suggesting that non-response bias was unlikely to affect the results.

Following data cleaning, 516 valid responses were retained for analysis. The responses exceed the recommended sample size for PLS-SEM and provide sufficient power to analyse complex models with mediation and moderation effects.

### 4.3 Measures

We used measures of all constructs adapted from previous research to ensure validity and reliability. All items used a five-point Likert scale (1 = strongly disagree, 5 = strongly agree).

AI adoption was assessed as employees’ integration of AI-enabled tools into their daily work through intelligent automation, AI-assisted decision support, and human–AI collaboration. Measurement items were adapted from technology acceptance studies (Venkatesh et al., 2012) and refined to capture employees’ routine interaction with AI-enabled work systems. Digital work intensity was measured using items reflecting workload, pace and digital work (Dettmers et al., 2016). Technostress was assessed using the scale developed by Tarafdar et al. (2019). Psychological resilience was measured using items from Luthans et al. (2007). Organisational support was measured using Eisenberger et al.’s (1986) scale. Wellbeing was measured using existing measures of psychological and emotional wellbeing.

First, the AI Adoption construct was further developed to focus on employees’ individual-level behaviours and extent of integration with AI tools in their daily work, instead of creating an AI Adoption composite score by aggregating three constructs: organisational implementation, perceived usefulness, and comfort. Items related to organisational deployment and perceived usefulness were eliminated to enhance construct purity. Second, the Employee Wellbeing construct was re-specified as subjective work-related psychological wellbeing, assessed by positive functioning, emotional balance, and satisfaction with work experiences. Broad mental health-related items, general happiness and motivational states were omitted to prevent conceptual overlap. Technostress was operationalised as a reflective construct measured using five items adapted from Tarafdar et al. (2019) and Ragu-Nathan et al. (2008). The items capture employees’ perceptions of technology-induced strain associated with AI-enabled work environments, including increased workload, complexity, uncertainty, and the need for continuous learning. All scales were translated and back-translated with the involvement of three HRM scholars and two HRM experts. The item clarity and relevance test was done in the pilot study with 42 IT employees. The content was validated through expert agreement and slight wording changes before the administration of the primary survey.

The measurement scales used in this study, along with their operational definitions, sources, number of items, and representative sample items, are presented in Table 1.

**Table 1:** Measurement of constructs.

Construct	Operational definition	Source	No. of items	Scale adaptation
AI adoption	Employees’ integration of AI-enabled tools into their daily work through intelligent automation, AI-assisted decision support, and human–AI collaboration.	Adapted from Venkatesh et al. (2012)	5	Refined to capture employees’ routine interaction with AI-enabled work systems.
Digital work intensity	Pace and volume of digitally mediated work characterised by continuous connectivity, multitasking, rapid information processing, and digital workload.	Dettmers et al. (2016)	5	Minor wording changes for AI-enabled workplaces.
Technostress	Psychological strain arising from AI-enabled work demands, including techno-overload, techno-complexity, and techno-uncertainty.	Tarafdar et al. (2019)	5	Modelled as a higher-order reflective construct.
Psychological resilience	Employees’ ability to adapt successfully and recover from challenges in AI-enabled work environments.	Luthans et al. (2007)	5	Contextualised for AI-enabled work settings.
Perceived organisational support	Employees’ perception that the organisation provides AI-related training, technical support, communication, and opportunities to develop AI competencies.	Eisenberger et al. (1986)	5	Adapted to reflect AI-specific organisational support.
Employee wellbeing	Work-related psychological wellbeing reflecting positive functioning, emotional balance, and satisfaction with work experiences.	Existing psychological wellbeing scales	5	Refined to measure work-related psychological wellbeing.

Source: Developed by the authors based on Venkatesh et al. (2012), Dettmers et al. (2016), Tarafdar et al. (2007), Ragu-Nathan et al. (2008), Luthans et al. (2007), and Eisenberger et al. (1986).

All items were measured using a five-point Likert scale ranging from 1 = Strongly Disagree to 5 = Strongly Agree. The measurement scales were adapted to reflect the context of AI-enabled work systems.

### 4.4 Data analysis

IBM SPSS and SmartPLS 4 were used for data analysis. SPSS was used for preliminary data analysis (descriptive statistics, checks for missing values and outliers). SmartPLS 4 was used to conduct Partial Least Squares Structural Equation Modelling (PLS-SEM), which is appropriate for complex models with multiple constructs, multiple mediators and moderators (Hair et al., 2011, 2014, 2017, 2019).

A two-step approach was followed. In the first step, the measurement model was assessed using indicator loadings, Cronbach’s alpha, composite reliability, average variance extracted (AVE), and discriminant validity (Fornell–Larcker criterion and HTMT ratio). The structural model was then evaluated using path coefficients, t-statistics, and *p*-values (bootstrapped with 5,000 resamples). Indirect effects were used to test mediation, and interactions to test moderation. We examined the model’s predictive power using *Q*^2^ values and used the Importance-Performance Map Analysis (IPMA) to determine managerial priorities (Ahmad and Afthanorhan, 2014; Ringle and Sarstedt, 2016); used the Importance-Performance Map Analysis.

### 4.5 Common method bias and non-response bias

We used Harman’s single-factor test to assess common method variance (CMV), and a single factor did not account for 50% or more of the variance, suggesting that CMV was not an issue (Podsakoff et al., 2012). A marker-variable technique was employed using a four-item scale measuring preference for routine work activities, which is theoretically unrelated to AI adoption, technostress, psychological resilience, organisational support, and employee wellbeing. Sample items included: “I prefer work tasks that follow a fixed routine” and “I feel more comfortable when work activities remain predictable.” The marker variable showed low correlations with the substantive constructs (*r* = 0.03–0.11), and the inclusion of the marker variable did not produce significant changes in the structural path coefficients, indicating that CMB was unlikely to threaten the findings. Procedural remedies included temporal separation through a two-wave design, psychological separation of predictor and outcome variables, anonymous participation, randomised item order, and assurance that there were no right or wrong answers.

### 4.6 Ethical consideration

The research was carried out in accordance with the established ethical considerations for research involving human subjects. The institutional ethics committee gave ethical approval via No. VIT/IECH/2025/16 IECH/ 15 February 2025/44. Involvement was voluntary, and all respondents were informed and gave their consent before data collection. The respondents were promised confidentiality and anonymity, and the data could only be used for academic purposes. Ethical approval was obtained from the Institutional Ethics Committee for Human Research (IECHR), VIT University, Vellore, India (Approval No. VIT/IECHR/2025/44; approved on 15 February 2025). Participants provided electronic informed consent before accessing the survey. No identifying information was retained in the final dataset; responses were stored on password-protected systems accessible only to the research team, and the de-identified dataset will be made available by the corresponding author upon reasonable academic request, subject to institutional ethical guidelines and participant confidentiality protections.

### 4.7 Demographic profile

Table 2 presents the demographic portrait of the respondents (*N* = 516) and provides an overview of the sample characteristics. The table shows that it is a diverse, experienced, and appropriate sample for investigating AI integration and the intensity of digital work in IT.

**Table 2:** Demographic profile of the respondents.

Demographic variable	Category	Frequency (*N*)	Percentage (%)
Gender	Male	298	57.80%
	Female	218	42.20%
Age group	Below 25 years	96	18.60%
	25–34 years	228	44.20%
	35–44 years	132	25.60%
	45 years and above	60	11.60%
Educational qualification	Undergraduate	104	20.20%
	Postgraduate	312	60.50%
	Doctorate	100	19.30%
Work experience	Below 2 years	82	15.90%
	2–5 years	176	34.10%
	6–10 years	158	30.60%
	Above 10 years	100	19.40%
Job position	Entry-level	168	32.60%
	Middle level	226	43.80%
	Senior-level	122	23.60%
Type of organisation	IT Services	304	58.90%
	Product-based IT	152	29.50%
	IT consulting	60	11.60%

Source: author’s creation.

## 5 Data analysis

PLS-SEM was selected because the study aimed to predict employee wellbeing and examine a complex model containing multiple mediators (Technostress and Psychological Resilience), a moderator (Perceived Organisational Support), and indirect effects, while making minimal distributional assumptions. The analysis was conducted using SmartPLS 4.0 with the path-weighting scheme, a maximum of 300 iterations, and a stop criterion of 1 × 10^−7^. Missing values were below 2% and were handled using the expectation–maximisation procedure before model estimation. Statistical significance was assessed through bootstrapping with 5,000 resamples and bias-corrected and accelerated (BCa) 95% confidence intervals. The revised manuscript now reports path coefficients, *t*-values, *p*-values, and bootstrapped confidence intervals for all direct and indirect effects. Inner VIF values ranged from 1.12 to 3.41, indicating no multicollinearity concerns. The moderation effect was estimated using the two-stage interaction approach, and conditional indirect effects were examined at low (−1 SD), mean, and high (+1 SD) levels of perceived organisational support. In addition, the index of moderated mediation was calculated and found to be non-significant (*β* = −0.006, 95% BCa CI [−0.018, 0.004]), indicating that the indirect effects did not vary significantly across levels of organisational support.

### 5.1 Measurement model

**Figure 2:**
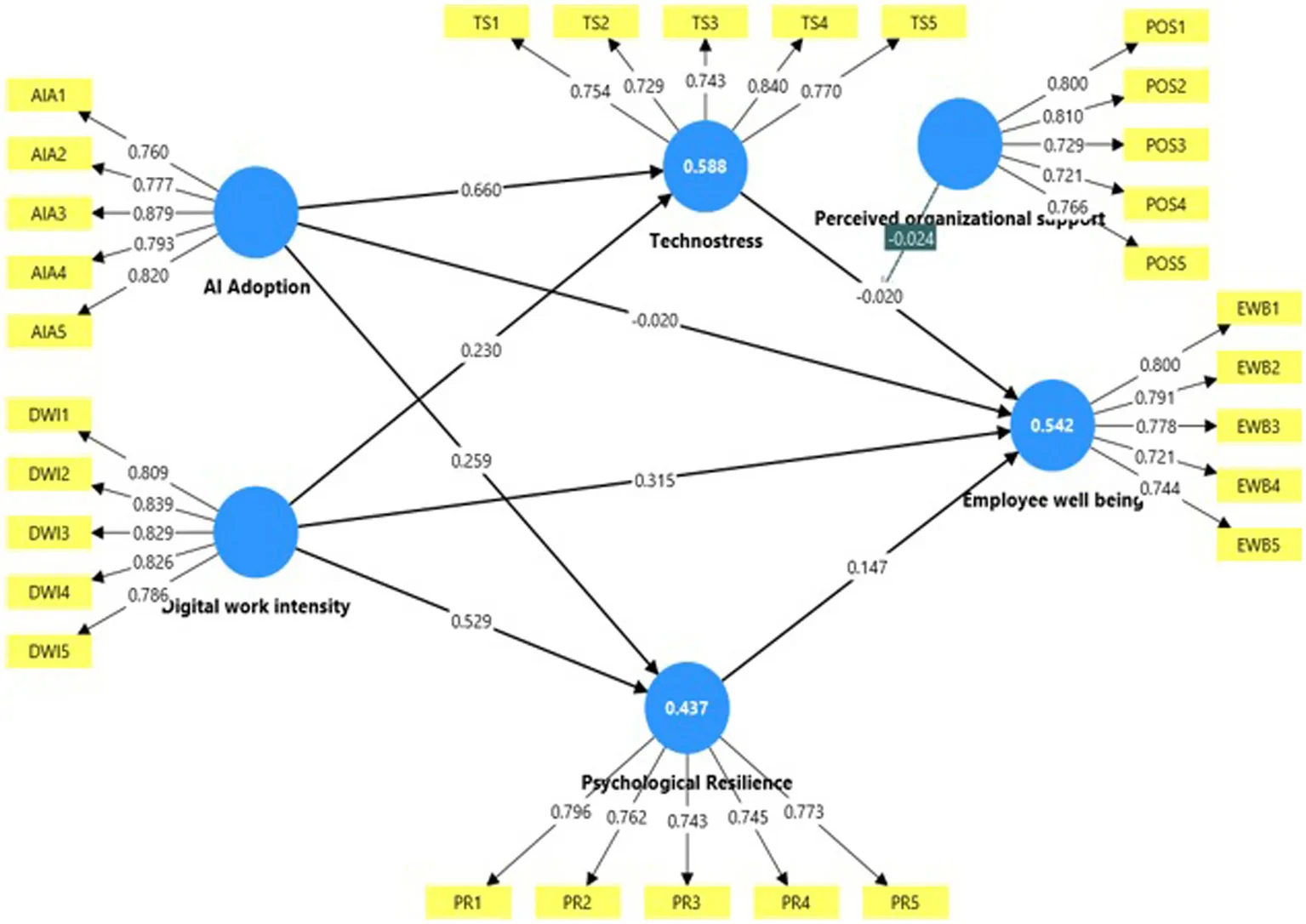
Measurement model.

The measurement model was evaluated using PLS-SEM guidelines (Hair et al., 2017, 2019). The reliability of indicators, internal consistency, convergent validity, discriminant validity, and multicollinearity were assessed. The loadings of all indicators were above the recommended value of 0.70. The reliability of the instrument’s items was supported by Cronbach’s alpha and composite reliability values exceeding 0.70. Convergent validity was proven as all AVEs were higher than 0.50. The Fornell–Larcker criterion (Fornell and Larcker, 1981) and the HTMT ratio were employed to assess discriminant validity, and both indicated that all values met the recommended limits. Furthermore, there were no multicollinearity issues because the variance inflation factor (VIF) values were all below 5. The overall results of the measurement model, as illustrated in Table 3 and Figure 2, indicated that reliability and validity were satisfactory; thus, it was appropriate for further analysis of the structural model.

**Table 3:** Constructs reliability and validity.

Construct	Item	Loading	*α*	ρA	CR	AVE	VIF
AI adoption	AIA1	0.760	0.865	0.866	0.903	0.651	1.879
	AIA2	0.777					2.033
	AIA3	0.879					2.901
	AIA4	0.793					2.024
	AIA5	0.820					2.356
Digital work intensity	DWI1	0.809	0.876	0.880	0.910	0.669	1.982
	DWI2	0.839					2.252
	DWI3	0.829					2.002
	DWI4	0.826					2.181
	DWI5	0.786					1.896
Technostress	TS1	0.754	0.826	0.828	0.878	0.590	1.668
	TS2	0.729					1.558
	TS3	0.743					1.631
	TS4	0.840					3.203
	TS5	0.770					2.714
Psychological resilience	PR1	0.796	0.822	0.824	0.875	0.584	1.880
	PR2	0.762					1.732
	PR3	0.743					1.629
	PR4	0.745					1.597
	PR5	0.773					1.732
Perceived organisational support	POS1	0.800	0.824	0.827	0.876	0.587	2.201
	POS2	0.810					2.283
	POS3	0.729					1.408
	POS4	0.721					1.568
	POS5	0.766					1.634
Employee wellbeing	EWB1	0.800	0.825	0.825	0.877	0.589	1.799
	EWB2	0.791					1.917
	EWB3	0.778					1.742
	EWB4	0.721					1.443
	EWB5	0.744					1.637

Source: author’s creation.

#### 5.1.1 Fornell–Larcker criterion

The Fornell–Larcker criterion (Fornell and Larcker, 1981) confirms discriminant validity, as shown in Table 4. Each construct’s AVE exceeded its correlations with the other constructs, suggesting that each construct explained more variance in its indicators than the other constructs did.

**Table 4:** Fornell–Larcker criterion.

Variables	AI adoption	Digital work intensity	Employee wellbeing	Perceived organisational support	Psychological resilience	Techno stress
AI adoption	0.807					
Digital work intensity	0.329	0.818				
Employee wellbeing	0.287	0.635	0.767			
Perceived organisational support	0.394	0.582	0.657	0.766		
Psychological resilience	0.433	0.614	0.583	0.636	0.764	
Technostress	0.736	0.446	0.373	0.445	0.569	0.768

Source: author’s creation.

#### 5.1.2 HTMT criterion

The data in Table 5 also support discriminant validity. The results indicated that all HTMT values were below the 0.90 threshold recommended by Henseler et al. (2015), which signalled that these constructs were empirically distinct and represented different domains.

**Table 5:** HTMT criterion.

Variables	AI adoption	Digital work intensity	Employee wellbeing	Perceived organisational support	Psychological resilience	Technostress
AI adoption						
Digital work intensity	0.374					
Employee wellbeing	0.364	0.743				
Perceived organisational support	0.479	0.678	0.779			
Psychological resilience	0.509	0.72	0.703	0.757		
Technostress	0.867	0.52	0.48	0.549	0.685	

Source: author’s creation.

### 5.2 Structural model

**Figure 3:**
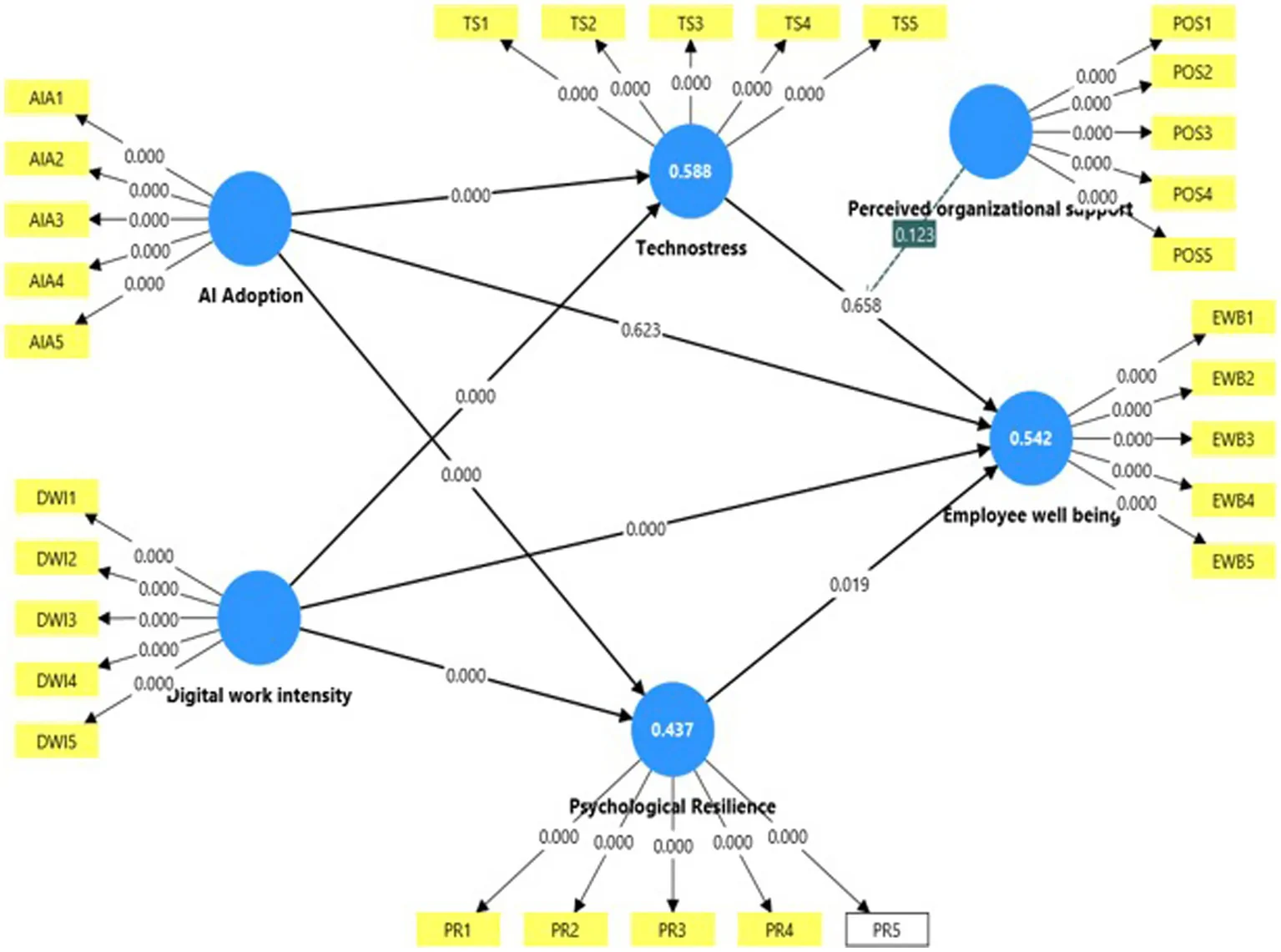
Structural model.

To test the hypothesised relationships between constructs, SmartPLS 4 was used to test the structural model. The analysis included estimating path coefficients, t-statistics, and *p*-values via bootstrapping (5,000 resamples) to assess the importance of relationships (Hair et al., 2017). The model’s explanatory power was measured by the *R*^2^ values for the endogenous constructs, which indicate the proportion of variance explained by the predictor variables. The effect size (*f*^2^) was analysed to examine the effects of each exogenous construct. Predictive relevance was measured using *Q*^2^ values obtained from the blindfolding process. Also, VIF values were used to check multicollinearity. Indirect paths and interaction terms were used to analyse mediation and moderation effects, covering all aspects of the structural model. The structural model with path coefficients is illustrated in Figure 3

#### 5.2.1 *F*-squared

Table 6 shows that the effect size (*f*^2^) measures the contribution of each exogenous construct to the *R*^2^ of the endogenous variables. According to the findings, we can interpret as follows: In the case of Technostress, AI Adoption (*f*^2^ = 0.944) has a larger effect size, indicating that it is a dominant predictor of technostress. Conversely, Digital work intensity (*f*^2^ = 0.114) exhibits a medium-weak effect, indicating it still plays a significant role, though not strongly. In the case of psychological resilience, the effect size for Digital work intensity (*f*^2^ = 0.444) is large, indicating a significant impact on resilience. Meanwhile, AI Adoption (*f*^2^ = 0.107) shows a small-to-medium effect, indicating a slight contribution. Digital work intensity (*f*^2^ = 0.112) and Perceived Organisational Support (*f*^2^ = 0.157) demonstrate low-to-medium effects, with a slight but more pronounced effect in the latter. The effect of psychological resilience (*f*^2^ = 0.020) is quite small, whereas technostress (*f*^2^ = 0.000) has no effect. The POS × technostress (*f*^2^ = 0.005) also shows a weak effect, corroborating the weak moderation. In general, the findings indicate that AI Adoption is a powerful contributor to technostress. In contrast, Digital work intensity can boost psychological resilience, moderating the relationship between the two.

**Table 6:** *F*-squared.

Predictor → outcome	Technostress	Psychological resilience	Employee wellbeing
AI adoption	0.944 (large)	0.107 (small–medium)	0.000 (none)
Digital work intensity	0.114 (medium)	0.444 (large)	0.112 (medium)
Psychological resilience	—	—	0.020 (small)
Technostress	—	—	0.000 (none)
Perceived organisational support	—	—	0.157 (medium)
POS × Technostress	—	—	0.005 (negligible)

Source: author’s calculation.

#### 5.2.2 *R*-squared

Table 7 presents the R-square (*R*^2^) values, which indicate how well the structural model explains the endogenous constructs. The model’s *R*^2^ is 0.542 (adjusted *R*^2^ = 0.536), indicating that the predictors of employee wellbeing explain about 54.2% of the variance, which is moderate to strong. The *R*^2^ for psychological resilience is 0.437 (adjusted *R*^2^ = 0.435), indicating that 43.7% of its variance is explained by AI adoption and digital work intensity, suggesting a moderate level of explanatory power. Similarly, technostress exhibits the highest *R*^2^ of 0.588 (adjusted *R*^2^ = 0.586), indicating that 58.8% of its variation can be explained by the independent variables, suggesting substantial explanatory power. The similarity between the *R*^2^ and adjusted *R*^2^ values across all constructs indicates that the model is stable and unbiased. All in all, the findings support the model’s strong predictive ability, especially in the context of digital work and in explaining factors such as technostress and employee wellbeing.

**Table 7:** *R*-squared.

Variables	R-square	R-square adjusted
Employee wellbeing	0.542	0.536
Psychological resilience	0.437	0.435
Technostress	0.588	0.586

Source: author’s calculation.

#### 5.2.3 *Q*-square

Table 8, *Q*^2^, predict outcomes shows that the model has excellent predictive performance across all endogenous constructs. The *Q*^2^ values for Psychological Resilience (0.434), Technostress (0.583), and Employee Wellbeing (0.526) are all positive and significantly greater than zero, indicating that the model has some predictive power out of sample. Based on the generally accepted thresholds, *Q*^2^ values above 0.35 indicate high predictive relevance, which applies to all three constructs in this model. Technostress has the greatest predictive validity (*Q*^2^ = 0.583), followed by Employee Wellbeing (*Q*^2^ = 0.526) and psychological resilience (*Q*^2^ = 0.434). The prediction errors also support this conclusion. The RMSE (0.649–0.761) and MAE (0.480–0.556) values are not very high, and the constructs exhibit relatively low deviation, indicating little variation between the predicted and actual values. The findings indicate that the model produces consistent, sound forecasts. On balance, the results indicate that the structural model not only describes the relations effectively but also predicts the unobserved data well. It enhances the model’s strength and practical relevance, especially in understanding how AI adoption and work intensity shape digital impact on employee outcomes through mediating processes.”

**Table 8:** *Q*-squared.

Variables	*Q*^2^ predict	RMSE	MAE
Psychological resilience	0.434	0.761	0.556
Technostress	0.583	0.649	0.507
Employee wellbeing	0.526	0.699	0.480

Source: author’s creation.

#### 5.2.4 Hypothesis testing—interpretation

Table 9 shows the results of the structural model. There was no significant direct relationship between AI use and employee wellbeing, but there was a positive direct relationship between digital work intensity and employee wellbeing. AI adoption and digital work intensity had significant effects on both technostress and psychological resilience, with both positive and negative impacts, suggesting that AI can create both stressors and resource-building processes.

**Table 9:** Hypotheses testing (direct effects).

Hypothesis	Path	*β* (O)	SD	*t*-value	*p*-value	Decision
H1	AI adoption → employee wellbeing	0.005	0.034	0.151	0.880	Not supported
H2	Digital work intensity → employee wellbeing	0.388	0.056	6.896	0.000***	Supported
H3	AI adoption → technostress	0.660	0.030	21.871	0.000***	Supported
H4	Digital work intensity → technostress	0.230	0.045	5.116	0.000***	Supported
H5	AI adoption → psychological resilience	0.259	0.036	7.247	0.000***	Supported
H6	Digital work intensity → psychological resilience	0.529	0.051	10.336	0.000***	Supported
H7	Technostress → employee wellbeing	−0.020	0.045	0.442	0.658	Not supported
H8	Psychological resilience → employee wellbeing	0.147	0.063	2.345	0.019**	Supported

Source: author’s calculation. **p < 0.01; ***p < 0.001.

As for the strain pathway, no significant effect of technostress on employee wellbeing was found, nor was there support for technostress’s mediating effects. By contrast, psychological resilience was strongly and positively associated with employee wellbeing and played a significant role in the relationships among AI adoption, digital work intensity, and employee wellbeing. The results indicate that workplace responses to AI-enabled work systems is more strongly motivated by resource-related than strain-related mechanisms.

Perceived organisational support had a very significant positive impact on employee wellbeing. Still, the moderating effect was not significant when introduced into the regression analysis of the relationship between technostress and wellbeing. This suggests that organisational support acts more as a direct organisational resource than as a buffer to technology stress.

The findings suggest that AI-supported work systems are associated with both demand-related and resource-related psychological processes, and that the work system’s demands on employees are more strongly determined by adaptive resources, such as psychological resilience, than by technology-related demands.

#### 5.2.5 Mediation analysis

Table 10 presents a mediation analysis of the indirect effects of AI Adoption and Digital Work Intensity on Employee Wellbeing via Technostress and Psychological Resilience. First, the mediation of Technostress between AI Adoption and Employee Wellbeing is not significant (0.013, *p* = 0.66), indicating that technostress does not mediate this association. The result implies that, despite the strong impact of AI adoption on technostress, the stress does not translate into changes in employee wellbeing. Likewise, the relationships between Digital work intensity, technostress, and Employee Wellbeing are not significant (*β* = −0.005, *p* = 0.664). Therefore, technostress is not an effective mediator in the model, which supports previous results indicating that its influence on wellbeing is not significant.

**Table 10:** Mediation analysis (specific indirect effects).

Hypothesis	Indirect path	*β* (O)	SD	*t*-value	*p*-value	Decision
H9	AI adoption → technostress → employee wellbeing	−0.013	0.029	0.441	0.660	Not supported
H11	AI adoption → psychological resilience → employee wellbeing	0.038	0.018	2.115	0.034**	Supported
H10	Digital work intensity → technostress → employee wellbeing	−0.005	0.010	0.435	0.664	Not supported
H12	Digital work intensity → psychological resilience → employee wellbeing	0.078	0.034	2.289	0.022**	Supported

Source: author’s calculation. **p < 0.01.

In contrast, psychological resilience is significant. The Adoption of AI indirectly affects employee wellbeing through psychological resilience (*β* = 0.038, *p* = 0.034). The use of AI will increase psychological resilience, thereby improving employees’ wellbeing. Similarly, the two-way relationship between Digital work intensity and employee wellbeing is statistically significant (χ2 = 0.078, *p* = 0.022), indicating that resilience mediates this relationship. In general, the findings emphasise that psychological resilience is a significant explanatory factor, but technostress does not mediate relationships. These results indicate that positive adjustment potentials outweigh stress-related pathways, helping explain the effects of digital and AI-based work environments on employee wellbeing.

#### 5.2.6 Moderation analysis

Table 11 presents the moderation analysis examining the role of Perceived Organisational Support (POS). The direct effect of POS on employee wellbeing was positive and significant (*β* = 0.373, *p* = 0.001), indicating that employees who perceive higher organisational support report greater wellbeing. This finding is consistent with prior research suggesting that supportive organisational environments positively affect employees’ psychological outcomes.

**Table 11:** Moderation analysis.

Path	Original sample (O)	Sample mean (M)	Standard deviation (STDEV)	*T* statistics (|O/STDEV|)	*p-*values	Decision
Perceived organisational support → employee wellbeing	0.373	0.371	0.053	6.989	0.000	Supported
Perceived organisational support × technostress → employee wellbeing	−0.024	−0.022	0.016	1.541	0.123	Not supported

Source: author’s calculation.

**Figure 4:**
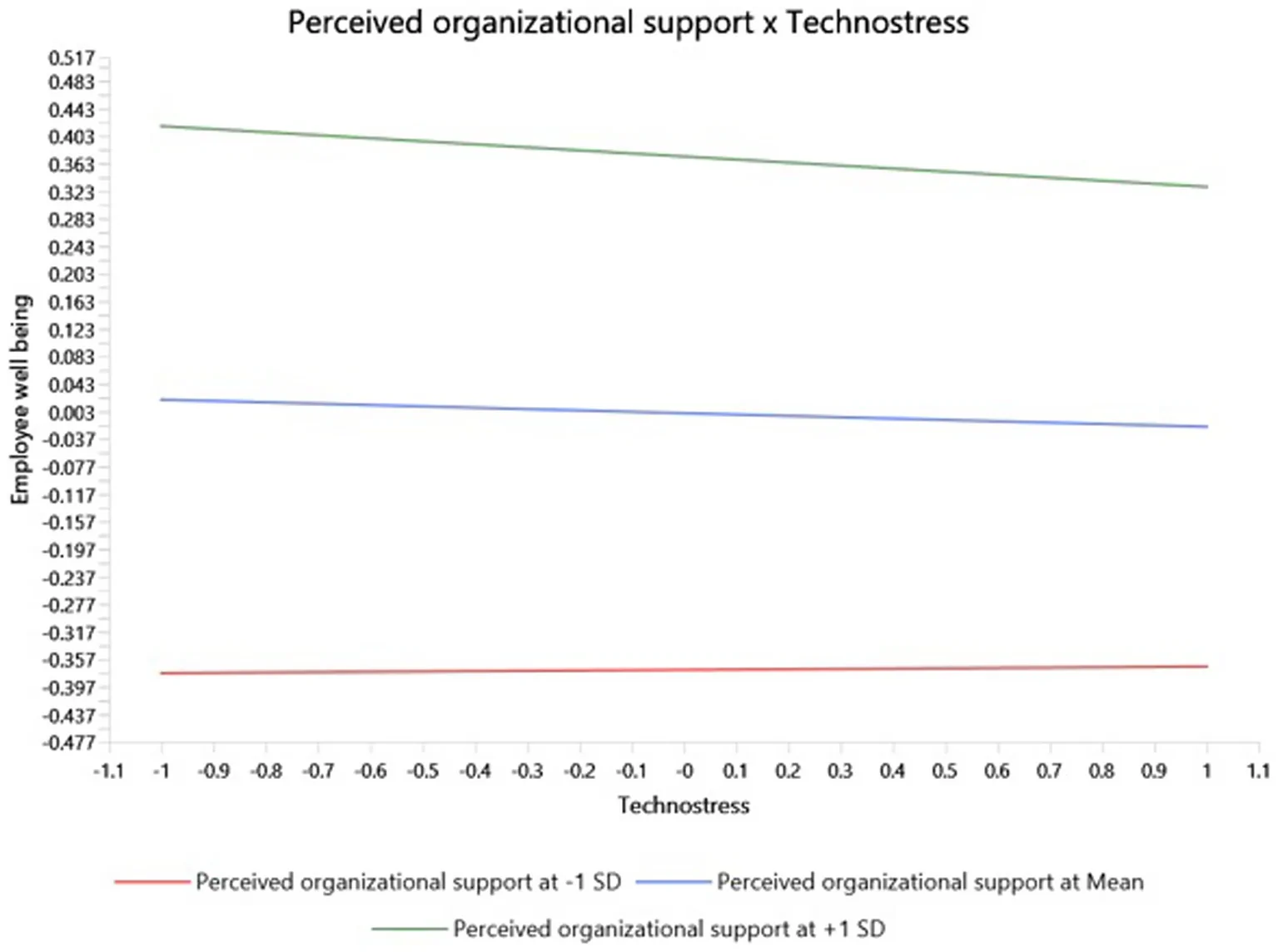
Moderation analysis.

However, the interaction effect between POS and Technostress was not significant (*β* = −0.024, *p* = 0.123). Thus, POS did not moderate the relationship between technostress and employee wellbeing. While organisational support directly enhances employee wellbeing, it does not appear to buffer the adverse effects of Technostress in AI-enabled work environments. These findings suggest that organisational support functions primarily as a direct organisational resource rather than a stress-buffering mechanism. Figure 4 illustrates the moderation effect of POS on the relationship between technostress and employee wellbeing.

### 5.3 IPMA analysis

**Figure 5:**
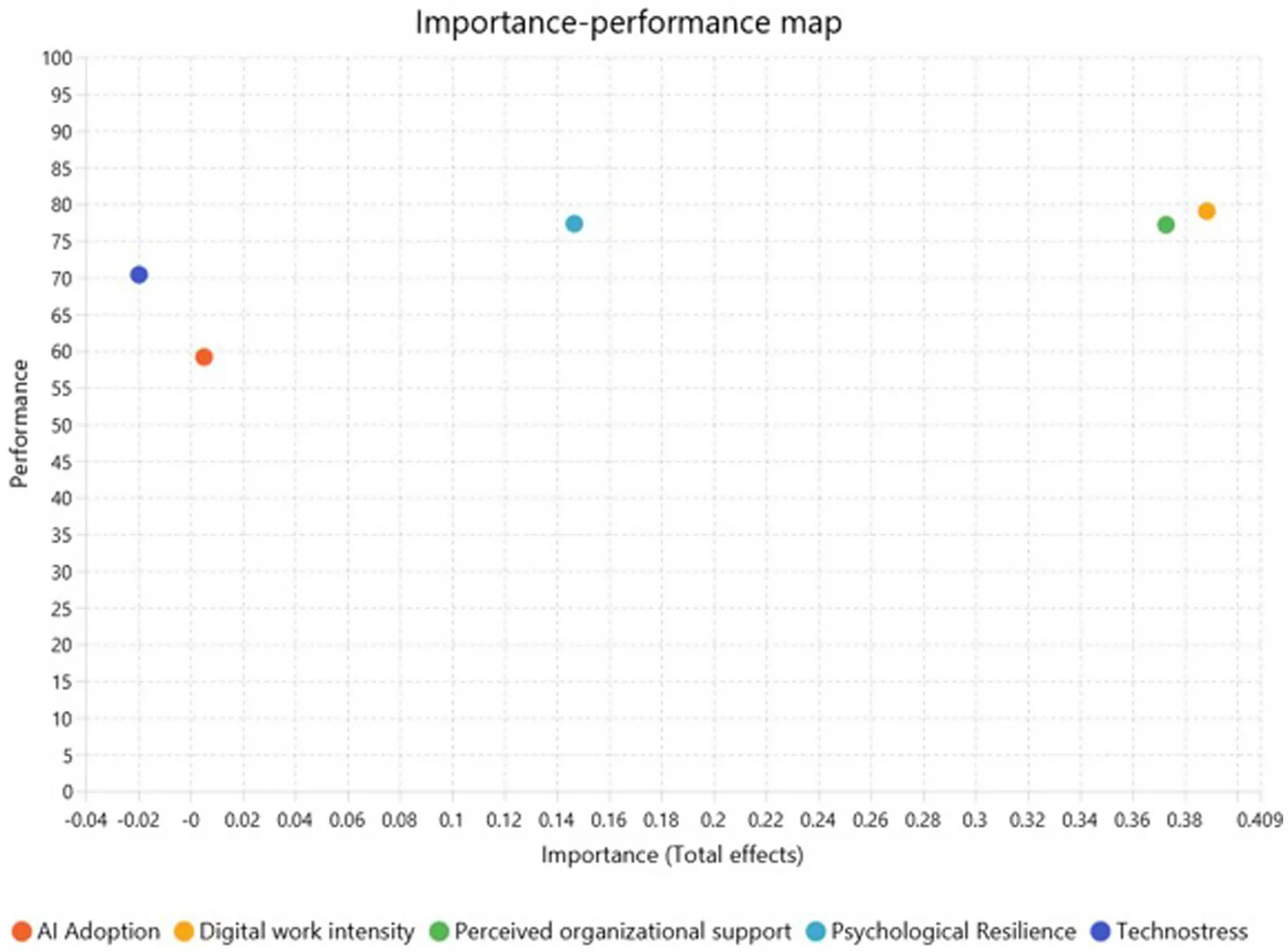
Importance–performance map analysis (IPMA).

To determine managerial priorities for improving employee wellbeing, the Importance–Performance Map Analysis (IPMA) was conducted (Ahmad and Afthanorhan, 2014; Ringle and Sarstedt, 2016). The latent variable importance and performance scores are presented in Table 12, while the graphical importance–performance map is shown in Figure 5. Results suggest that psychological resilience and perceived organisational support are both important and effective and should therefore be seen as key factors in improving employee wellbeing. Within the model, digital work intensity also performs well. By contrast, AI adoption and technostress are comparatively low, offering opportunities for managerial intervention. The results suggest that, alongside implementing AI, organisations can improve workers’ resilience through ongoing education and supportive policies. Overall, IPMA findings underscore the need to strike a balance between technological progress and resources dedicated to employees in AI-enabled work environments (Ringle and Sarstedt, 2016).

**Table 12:** IPMA LV performance.

Variables	LV performance
AI adoption	59.134
Digital work intensity	79.021
Employee wellbeing	77.761
Perceived organisational support	77.160
Psychological resilience	77.309
Technostress	70.359

Source: author’s calculation.

## 6 Discussion

This study explored employees’ psychological responses in AI-infused workplaces, focusing on the impact of AI adoption and digital work intensity on employee wellbeing via competing strain and resource pathways. The results are based on the Job Demands–Resources (JD-R) model and the Conservation of Resources (COR) theory, thereby offering a more detailed insight into employee reactions to increasingly technology-mediated workplaces. The outcomes provide a basis for three key themes. The results can be analysed using the framework of Conservation of Resources (COR) theory, which focuses on the simultaneous processes of resource loss and resource gain. Initially, AI-enhanced work systems could utilize workers’ cognition and emotions by making the jobs more complicated, uncertain, and demanding of learning. Successful interactions with AI systems can also equip employees with new technologies and skills, new confidence in both technological and digital matters, developing new problem-solving skills, and building adaptive capacity. The strong indirect pathway also indicates that such resource-gain pathways may be important in impacting employee wellbeing, particularly through psychological resilience. The results not only show that AI can be a source of stress, but also a chance to create personal resources for employees as they adjust to intelligent technologies. The findings are consistent with COR theory, which proposes that successful adaptation may contribute to future resource gains. However, the present two-wave study does not directly test these dynamic processes.

This finding can be more precisely explained by the Conservation of Resources (COR) theory, which suggests that people work to acquire, preserve and defend valuable resources and that stress occurs when their resources are threatened or depleted. In the current study, the use of AI and the intensity of digital work were linked to higher levels of technostress, indicating that AI work systems could heighten cognitive load, uncertainty, and the requirement for lifelong learning, causing psychological resources to be taken up by the employees. In the present digitally skilled IT sample, however, non-significant relations between technostress and wellbeing suggest that the perceived loss of resources did not relate to poorer wellbeing. Psychological resilience, on the other hand, had a significant positive relationship with employee wellbeing, and acted as a mediator between AI adoption and digital work intensity. A COR perspective would be that, in an AI-enabled environment, employees can also enhance or develop their personal resources (confidence, adaptability, coping capacity, etc.). The findings thus suggest that adaptive personal resources can be a critical factor in sustaining wellbeing amid technology demands. That loss and gain of resources can happen at the same time, as COR theory claims.

### 6.1 AI-enabled work systems generate both demands and resources

“The findings support this: The AI workplace is challenging and offers opportunities. The spread of AI and digital work intensity was positively correlated with technostress, suggesting that the use of AI and digital work is linked to increased cognitive effort, technological complexity, learning requirements, and connectivity. The findings of this study corroborate prior studies suggesting that digital transformation is associated with new workplace pressures, meaning people are continually adapting to new working practices and technology.

In contrast, psychological resilience was positively associated with AI use and the intensity of digital work. The finding suggests that technologically advanced work environments are not only areas of employee needs but also places to learn, build skills, and develop adaptively. When employees incorporate AI into their work systems, they appear to be growing more confident and competent in addressing uncertainty and change. So, it’s important to take a holistic view of AI and its use in the workplace, rather than viewing it as a source of stress or productivity gains. Rather, they are intricate working environments that offer challenges and opportunities that go hand in hand.

This study contributes to the JD-R theory by demonstrating a strong correlation between work demands and AI-related resources. They also validate certain ideas in COR theory that gains and losses of resources can occur simultaneously. The results, therefore, highlight the need for a balanced view of AI-based work systems, recognising the challenges and opportunities of digital transformation.”

### 6.2 Psychological resilience is more influential than technostress in shaping employee wellbeing

One of the major results of the study is that psychological resistance has a greater effect on employee wellbeing than does technostress. While the use of AI and the intensity of digital work had a significant impact on Technostress, Technostress itself did not significantly influence employee wellbeing. Moreover, the mediation analysis indicated that technostress was not a significant mediator between technology-related work conditions and employees’ outcomes.

Psychological resilience, however, was found to be a significant predictor and mediator of employee wellbeing. Staff with greater adaptive capacity reported higher wellbeing, even in technologically challenging settings. The findings indicate that the outcomes of AI use in the workplace are not solely a function of technological demands but rather of employees’ capacity to adjust to them.

Another possible reason for the non-significant direct relationship between AI adoption and employee wellbeing is that AI adoption may not guarantee positive psychological outcomes. Rather, its impact seems to be contingent on workers’ coping abilities and psychological resilience. The fact that employees with higher psychological resilience and digital readiness might see AI as a chance to learn, build skills, and advance their careers, rather than a cause of stress, could be a significant factor. The impact of AI on employee wellbeing will likely be indirect, in that employees’ ability to manage successfully in AI-supported workplaces will not be impacted directly by the adoption of AI.

This discovery adds to the growing research that questions the largely stress-based explanation of digital work. The literature on technostress has largely examined the negative impacts of technology adoption. Yet, the current results suggest that resource development processes offer a more complete understanding of employees’ experiences in AI-enabled workplaces. Psychological resilience seems to be an important resource employees have to overcome technological challenges and turn them into opportunities for growth and development.

The findings from this group, from an employees’ psychological responses perspective, suggest that digital transformation can be achieved not only by alleviating the strain associated with technology but also by enhancing employees’ adaptive capabilities. Resilience could become a growing factor affecting employee productivity and health in the future as organisations roll out AI capabilities across their workflows. The results indicate that technostress did not negatively impact employee wellbeing, which might be due to the nature of the employees sampled. In general, workers in the Indian IT sector are highly digitally literate, have significant experience of working with technology-mediated work, and have been exposed to constant change in technology. Consequently, they might view AI-related demands as challenge demands, which facilitate learning, skill development, and job progress, but are not considered hindrance demands that prevent performance and decrease wellbeing. A JD-R perspective allows challenge demands to co-exist with positive motivational states if employees have adequate personal and organisational resources. The finding consequently indicates that the potential negative effects of technostress might be less pronounced in highly technological job populations than in populations with less technological preparation or less experience with rapidly changing technologies. This interpretation also provides insight into the reasons for the rise in technostress following the adoption of AI, and why there was not necessarily a significant negative impact on wellbeing.

### 6.3 Organisational support improves wellbeing but does not substitute for adaptive capabilities

“The third important finding concerns perceived organisational support. It is revealed that employee wellbeing is positively associated with organisational support, and that supportive working conditions are significant in the context of technological change. Caring for and being valued by their team members and colleagues in the workplace are also associated with positive experiences and good health.

Contrary to expectations, the moderating effect of perceived organisational support on the relationship between technostress and employee wellbeing was not found. The study found that organisational support is not necessarily a buffer against the strain caused by AI use in the workplace. Organisational support, however, appears to be more of a direct contextual resource, positively impacting employees’ wellbeing, irrespective of their Technostress levels.

The results show that organisational and personal resources may play different roles in digital work contexts. Employees’ outcomes are perceived to be positively influenced by organisational factors, while psychological factors (e.g., psychological resilience) appear more relevant in supporting employees’ coping with the technological demands. It highlights the importance of providing support for employees’ personal coping and adaptation resources, as well as for the organisational support resources and interventions.

Based on the summarised results, it is concluded that technological, personal, and organisational resources interact in employees’ adaptation to the working environment in which they work with AI. With the introduction of Artificial Intelligence and the digitisation of work, new challenges arise. Still, people who can adapt and have their organisations behind them are more likely to thrive in the digital world.

## 7 Theoretical contributions and implications

The study supports research on AI-enabled workplaces, employee wellbeing, and workplace digitalisation by enhancing understanding of how employees adjust to technology-mediated workplaces. The results indicate that the interaction between strain and resource processes, as conceptualised in the JD-R model and Conservation of Resources (COR) theory, influences employees’ reactions to work systems involving AI.

First, by showing that AI adoption and digital work intensity are both job demands and job resource-generating processes, the study contributes to the literature on AI-enabled workplaces. Previous research has tended to focus on either the strain or the productivity and learning aspects of AI, but the results here indicate that both are present simultaneously. The use of AI systems in the workplace not only adds complexity and ongoing learning needs but also contributes to psychological resilience by empowering employees to build their adaptive capabilities, thereby increasing technostress. This is the first study to show how the work characteristics of AI activities can trigger both health impairment and motivation processes.

Second, the study adds to the literature on employee wellbeing and technology adaptation by showing that the resource-based mechanisms have a greater effect on employee outcomes than the strain-based mechanisms. Technostress increased significantly with the adoption of AI and with work intensity associated with digital technology. Still, it did not influence employees’ wellbeing, nor did it mediate the relationship between technology-related work conditions and employee outcomes. Conversely, psychological resilience was found to be a key predictor and mediator of employee wellbeing. The results indicate that employees’ adaptive resources, rather than strain from using AI, are more important for positive employee wellbeing in such workplaces. The study thus builds on prior studies of technostress by emphasising resilience as an important mediator in coping with digital transformation in the workplace.

Third, the study has implications for organisational support studies by shedding light on the role of perceived organisational support in an AI-enabled work environment. In other words, perceived organisational support was a significant positive influence on employees’ wellbeing, but it was not a moderating variable regarding the effect of technostress on wellbeing. The results contradict the notion that organisational support always serves as a buffer to stress. Organisational support, however, seems to function mainly as a contextual resource that directly supports employee wellbeing, irrespective of employees’ Technostress levels. Findings also indicate that organisational resources are less important than personal resources (such as psychological resilience) in the coping process when facing technology-related demands.

In total, these contributions highlight the importance of employees’ psychological responses as the mechanism by which AI-supported work systems impact workplace outcomes rather than technology adoption. The study offers a more holistic explanation of how employee wellbeing is shaped in digitally transformed workplaces by creating a single framework that incorporates technological demands, personal adaptive resources, and organisational support, and by extending current debates on the human dimensions of workplace digitisation and the future of work.

### 7.1 Practical implications

The results have several implications for work systems organizations that use AI. Organisations need to offer structured AI-skills training and continuous learning programs that can help employees become more comfortable with using AI tools and mitigate the uncertainty surrounding technology change. Second, managers need to create easy-to-access technical support avenues and AI help desks so that employees can get help promptly when experiencing system-related issues. Third, there is a need to invest in interventions that increase employee resilience, such as stress-management workshops, peer-support networks, coaching, and psychological wellbeing programmes. Fourth, clear communication about the goals of AI implementation, impacts on roles, data use, and outcomes can help mitigate confusion and increase trust. Fifth, employees need to be engaged in the decision-making process for implementing AI, either by using feedback channels, trial, or through participatory design workshops, to boost acceptance and perceived control. Lastly, HR teams should routinely check for signs of technostress and employee wellbeing through surveys and then modify the nature of the work tasks, training programs, and support offered at various phases of AI adoption.

## 8 Research limitations and future directions

The results of this study are subject to several limitations that must be taken into account when interpreting the findings. First, the cross-sectional design does not allow causal inference and cannot measure the evolution of employee wellbeing in AI-enabled work systems. Longitudinal or time-lagged designs could be used in future studies to investigate the changes in technostress, psychological resilience and employee wellbeing as workers become more experienced with AI technology. The study has a significant drawback in that it employed a two-wave time-lagged design. Still, the dynamics of the Job Demands–Resources (JD–R) and Conservation of Resources (COR) processes were not fully captured. The levels of technostress, psychological resilience and resource gains or losses experienced by employees will likely change as they develop experience using work systems that incorporate artificial intelligence. Longitudinal and multi-wave designs should therefore be used in future research to explore the trajectory of these processes, and the potential for development of resilience to mitigate the longer-term impact of technology-related demands. Further multi-level research in various industries and in various contexts of AI readiness would also boost the generalisability of the results.

Secondly, the study was based on self-reported data gathered through a survey from employees in the Indian IT industry. While both procedural and statistical remedies were used to reduce common method bias, future studies may enhance the validity of the findings by using multiple data sources, such as supervisor ratings, organisational records, and objective performance measures. Furthermore, the industry and national contexts may limit the generalisability of the results.

Third, the study explored the two most frequently cited mechanisms between AI-integrated workplaces and employees’ wellbeing: Technostress and psychological resilience. Although these constructs give some insight into employees’ psychological responses, other explanations might also be applicable. There is scope for further research into employee outcomes, employee engagement, job satisfaction, digital fatigue, AI trust, and human–AI collaboration. Similarly, while POS was considered a contextual resource, other boundary conditions, such as leadership style, organisational culture, digital literacy, and change readiness, can contribute to a fuller understanding of employee reactions to digitalisation in the workplace.

Lastly, AI usage and digital workload were measured as general categories. Additionally, future studies could examine various aspects of AI application, varying degrees of algorithmic engagement, and various dimensions of digital work intensity to gain a more detailed picture of how characteristics of technology-related work affect employee wellbeing.

Within its scope, the study is important because it demonstrates the extent to which employees adapt to AI in the workplace and serves as a basis for further research on the human side of workplace digitalisation and the future of work.

## 9 Conclusion

The study investigated the relationships between the factors of AI adoption, digital work intensity, technostress, psychological resilience, perceived organisational support, and employee wellbeing in the Indian IT industry through a two-wave time-lagged PLS-SEM design. The results show that the use of AI was not significantly related to workers’ wellbeing in the direct pathway, while digital work intensity was positively related to wellbeing. In summary, it could be said that the use of AI and the intensity of digital work were positively correlated with technostress and psychological resilience, suggesting that the use of AI in the workplace is a process of strain and resources. Technostress was not, however, significantly related to employees’ wellbeing, nor was it a mediator between the predictor variables and wellbeing. In contrast, psychological resilience had positive links to wellbeing and significant indirect links to the relationships with AI adoption and digital work intensity. There was a positive relationship between P.O.S and employee wellbeing; however, the interaction between P.O.S and technostress was not significant. Overall, the findings indicate that resource-based mechanisms, specifically psychological resilience, are more applicable than the strain pathway that was tested in understanding the wellbeing of the IT workers in this sample who are digitally skilled.

The study fills a gap in the literature on employee wellbeing, workplace digitalisation, and the future of work by demonstrating that employees’ psychological responses is an important pathway through which AI-enabled work systems affect workplace outcomes. The results build on previous research by illustrating the possibility of overcoming the strain-based processes in digitally intensive work settings through resource-building processes. In addition, the findings indicated that perceived organisational support serves as a direct organisational support service to increase employees’ wellbeing, rather than merely as a buffer against technology-related stress.

The study reveals that there was no significant direct relationship between employee wellbeing and the use of AI, nor was the mediation of technostress supported. The use of AI and digital work intensity were, however, positively related to psychological resilience, which, in turn, was positively related to psychological wellbeing at work. These results indicate that adaptation to AI-based work systems could be more related to the cultivation of adaptive personal resources than to the decrease of technology-induced strain. The results should therefore be considered as evidence of associational relationships, and not causal effects, because the design was an observational study with a time lag. Put together, it provides insights into employee responses to AI in the workplace by illustrating that intelligent technologies can create technological needs and opportunities for resource development, and that resilience is a critical pathway for employees to sustain positive psychological functioning in digitally driven work environments.

In practice, the results emphasise the need to couple technology investments with employee interventions. While technology is a critical piece of the puzzle, it’s also important to consider investments in initiatives that build employee resilience, ongoing learning, and adaptability. While supportive organisational practices remain significant, it is crucial to build employees’ ability to work effectively with technological change to succeed in AI-infused workplaces.

In summary, the study highlights the crucial need to involve employees in the digital transformation process. The changing nature of work with AI means it’s more important than ever to understand how employees will respond to an AI-driven environment and how best to support them as they navigate it, ensuring their wellbeing and the long-term success of the organisation.

## Data Availability

The raw data supporting the conclusions of this article will be made available by the authors, without undue reservation.
